# The Effects of Two Selected Single Nucleotide Polymorphisms of the Fatty Acid Synthase Gene on the Fat Content and Fatty Acid Profile of Cow’s Milk from the Polish Holstein–Friesian Red-and-White Breed versus Two Polish Red-and-White and Polish Red Conservation Breeds Kept in Poland

**DOI:** 10.3390/ani14152268

**Published:** 2024-08-04

**Authors:** Paulina Przybylska, Marian Kuczaj

**Affiliations:** Institute of Animal Breeding, Wroclaw University of Environmental and Life Sciences, ul. Chelmonskiego 38C, 50-375 Wroclaw, Poland

**Keywords:** FASN, SNPs, milk, fatty acids, milk fat, cattle

## Abstract

**Simple Summary:**

The composition of milk fat is of interest to milk producers and consumers. Unsaturated fatty acids are important fats in the human diet, and their profile is influenced by the single-nucleotide polymorphisms (SNPs) of the FASN gene. This study aimed to determine the relationship of two selected SNPs and their genotypic variants with the fat content and fatty acid profile of milk from Polish Red-and-White (ZR), Polish Red (RP), and Polish Holstein–Friesian Red-and-White (RW) breeds. The frequencies of the genotypes of the SNPs were also determined for each breed included in this study. This study enabled the determination of cattle breeds (each with its specific genotype of a given SNP), which proved to be more conducive to the production of health-promoting milk, as evidenced by the fat and fatty acid content of the milk. It was found that, for one SNP, the RP breed (with a specific genotype) exhibited favorable characteristics, while in the case of the other SNP, the RP, ZR, and RW breeds (each with their specific genotypes) demonstrated favorable attributes. This information can be used in cattle-breeding efforts to improve the quality of milk produced.

**Abstract:**

Fatty acid synthase (FASN) is a metabolic enzyme responsible for the synthesis of fatty acids in milk and meat. The SNPs g.841G/C and g.17924A/G of the FASN gene significantly influence the fat and fatty acid content of milk from cows of various breeds. Therefore, these SNPs were selected for this study. This study aimed to analyze the relationship of SNPs and their genotypes with the fat content and fatty acid profile of milk from Polish Red-and-White (ZR), Polish Red (RP), and Polish Holstein–Friesian Red-and-White (RW) cows. Milk samples were obtained during a milking trial. SNP genotyping was performed using the real-time PCR (HRM) method. It was shown that SNPs (with specific genotypes) were significantly associated with the presence of fatty acids such as C18:1n9t and C18:2n6c in milk. In addition, it was found that the milk fat from the ZR (genotypic variant A/G, AA) and RP (genotypic variant GG, A/G) breeds often exhibited a more attractive fatty acids profile than the milk fat from RW cows. This information can be used by both cattle breeders and people interested in consuming functional foods.

## 1. Introduction

The aim of breeding livestock (including cattle) is to obtain the best possible production characteristics resulting from a combination of animal selection and the use of molecular genetics methods involving candidate genes and DNA sections, which are referred to as quantitative trait loci (QTLs) [1]. Genetic variability (polymorphism) in the selection process serves to increase the productive characteristics of livestock and it is associated with the evolutionary potential of a population, which is usually higher for a population characterized by greater genetic variability [2].

FASN (fatty acid synthase) is a homodimeric enzyme that is responsible for the *de novo* regulation of long-chain fatty acids (LCFA) biosynthesis. FASN, which is present in mammals, is a metabolic enzyme that also plays an important role in embryonic development [3,4]. FASN is essential for the elongation of fatty acids in the process of *de novo* synthesis [5,6]; it is a cytosolic enzyme that catalyzes the *de novo* formation of palmitate from acetyl-CoA and malonyl-CoA in the presence of NADPH [7,8,9]. In addition, FASN determines the synthesis of saturated fatty acids (SFAs) and unsaturated fatty acids (UFAs) [6]. The FASN gene (GenBank accession AF285607) was mapped on the longer arm of chromosome 19 (BTA19) at 19q22 [10]; the bovine FASN gene is 19,760 bp long and consists of 42 exons and 41 introns [11].

Fat biosynthesis is a pathway involved in the metabolism of cow’s milk and is regulated by many genes [12]. Milk fat is a very important feature in dairy cattle breeding and the dairy industry [13]; it significantly determines the nutritional value of milk [3]. Milk fat is most often found in the form of triacylglycerols (TGs), which are formed from a glycerin molecule bound to three fatty acids [14]. TGs are synthesized from more than 400 fatty acids and account for more than 95% of milk fat. In addition to triglycerides, fat consists of diacylglycerol, phospholipids, and free fatty acids. The fat content of cow’s milk is, on average, 3–4% [15]. Fatty acids from C4:0 to C14:0 (short- and medium-chain fatty acids, which constitute two-thirds of milk fat [16]) and some C16:0 fatty acids are synthesized *de novo* in the mammary gland. Other C16 acids and acids with more than C18 LCFAs are absorbed from the blood [17].

The presence of a large proportion of saturated fatty acids (SFAs), such as myristic acid (C14:0) and palmitic acid (C16:0), is a risk factor for cardiovascular disease [18,19]. Conversely, desirable fats are those rich in monounsaturated fatty acids (MUFAs) [20]. For example, oleic acid (C18:1n9) lowers LDL levels while maintaining HDL levels at a level beneficial to health [19]. In addition, it is believed that MUFAs play an important role in reducing metabolic syndrome [21]—a group of various coexisting factors that increase the likelihood of cardiovascular system diseases [22].The fat content and fatty acid profile of the milk and meat from various cattle breeds are significantly influenced by the SNPs found in the FASN gene: g.841G/C (rs41920005) and g.17924A/G (rs41919985) [8,11,21,23,24,25,26,27,28,29,30,31,32,33].

In Poland, the most important breed of dairy cattle is a high-yield breed called the Polish Holstein–Friesian breed. There are two color varieties. According to data taken from the Polish Federation of Cattle Breeders and Milk Producers as of 31 December 2023, the Red-and-White (RW) variety, the subject of our research, constitutes only 3.50% (an average of 28,326 cows) of the dairy cows covered by the performance assessment of dairy cows in Poland. Even smaller percentages are found for ZR (the Polish Red-and-White breed) cows (0.50%; an average of 4059 cows) and RP (the Polish Red breed) (0.38%; an average of 3079 cows). In 2023, the ZR, RP, and RW cows were characterized by the following average yields, respectively (for a lactation period of 305 milking days): (1) milk—4223 kg, 3345 kg, and 8418 kg; (2) fat—4.14%, 4.26%, and 4.16%; (3) protein—3.27%, 3.37%, and 3.44%; and (4) fat + protein—313 kg, 256 kg, and 640 kg. The average lengths of use of the tested cows in 2023 were 6.56 years (ZR breed), 6.84 years (RP breed), and 3.17 years (RW breed) [34]. In 2019, the number of ZR cattle covered by the protection of genetic resources in Poland was 3378 animals (326 herds of animals). In turn, the number of RP cattle covered by the protection of genetic resources in the milk assessment was 2513 animals (257 herds of animals) [35,36] (Figures S1 and S2 in the Supplementary Materials).

The aim of this study was to determine the effect of the SNPs g.841G/C and g.17924A/G of the FASN gene (along with their genotypic variants) on the fat content and fatty acid profiles of the milk from cows in the three breeds studied. In addition, the frequencies of individual variants of the abovementioned SNP genotypes in all three cattle breeds were determined. Two of these breeds are included in conservation breeding programs in Poland: ZR and RP. The third breed included in the experiment was the RW breed. The results obtained may provide a basis for expanding and continuing research on the potential function of the analyzed SNPs of the FASN gene as genetic markers.

## 2. Materials and Methods

### 2.1. Animal Material

The research material consisted of 485 milk samples obtained from three cow breeds: Polish Red-and-White (ZR, *n* = 95), Polish Red (RP, *n* = 148), and Polish Holstein–Friesian Red-and-White (RW, *n* = 242). The milk was collected in the summer season (July 2019) and was obtained during a trial milking procedure (using the A4 and AT4 methods). The A4 and AT4 methods are similar methods for assessing dairy utility and are accepted by the international organization of ICAR (the International Committee for Animal Registration). The A4 method involves trial milkings every 4 weeks (a minimum of 11 milkings per year). This method uses a collective milk sample. The collective milk sample is a representative sample comprising all milkings carried out within 24 h (2 or 3milkings) and is intended for milk quality assessment analyses. In order for the collective sample to be representative, an equal volume of milk should be collected from each milking during the day. In the AT4 method, trial milkings are also carried out every 4 weeks (a minimum of 11 milkings per year), but they are conducted alternately; cows are milked in the morning in a specific month and in the evening in the next month, and so on. With this method, the milk sample intended for analysis is only collected via 1 milking per day (in a given month in the morning and the next month in the evening, and so on) [37]. The cows were fed according to a traditional barn–pasture system (PMR: partially mixed rations in a barn, with pasture-season grazing). The farms (*n* = 17) from which the cows came were located in northeast, southwest, and southern Poland and were included in the assessment of the usage value of dairy cattle. The average air temperature in the areas where the experimental cow herds were kept was 18.5 °C (the highest recorded average temperature was 20.3 °C, while the lowest average temperature was 17.1 °C). The range of these temperatures was optimal for the milked cows.

Due to the fact that the milk samples were obtained during trial milkings (performed regularly according to a specific schedule by a qualified zootechnician), there was no need to seek approval from the Local Ethics Committee for the procedure used to obtain the research material. The animals included in the study were not subjected to new stress factors.

The conditions for transporting the obtained samples to the laboratory and the temperature conditions for their storage until the determination of their fat content and DNA isolation were described in a previous article [38].

The somatic cell count (SCC) in the tested milk did not exceed 400,000/mL. The cows from which the milk was obtained were in good health and were kept in good welfare conditions. Based on information obtained from the animals’ breeders, it was found that at the time of collecting the milk samples, the cows were not sick and were not taking any medications. The herds of cows included in the study were under constant medical and veterinary care.

### 2.2. Milk Analyses

The milk samples were analyzed for their fat content using an infrared milk analyzer 150 (Bentley Instruments Inc., Chaska, MN, USA), while the somatic cell count (SCC) was evaluated using a Somacount 150 (Bentley Instruments Inc.).

The fatty acid profile was determined using a gas chromatograph, the Agilent Technologies 7890A GC System, fitted with a flame-ionization detector (Santa Clara, CA, USA). The fat was extracted from the milk using the Folch method [39]. Esterification was carried out with potassium hydroxide (KOH) in a mixture of methanol and hexane. The method for determining individual fatty acid esters and the temperature and time conditions for their separation were described previously by Przybylska and Kuczaj [38]. Agilent ChemStation no. B.03.02. (Agilent Technologies) software was used to identify the particular esters of the fatty acids.

The percentages of 28 fatty acids in the milk fat of the breeds included in this study were determined. The classification of all fatty acids thus determined was previously described by Przybylska and Kuczaj [38]. In addition, the percentage of all detected trans fatty acids (C18:1) was determined. The C15:1 and C18:2n6t acids were not included in further analyses due to their very low content in milk fat. However, the total amounts of all marked SFAs and all marked UFAs were considered. The sum of the total SFA and UFA contents was also analyzed.

### 2.3. Isolation of Genomic DNA and SNP Genotyping

To isolate genomic DNA from the milk, a Sherlock AX kit (A&A Biotechnology, Gdańsk, Poland) was used according to the manufacturer’s instructions. The concentration and purity of the DNA isolates were then determined using a NanoDrop 2000c spectrophotometer (Thermo Fisher Scientific, Wilmington, DE, USA).

The genotyping of single nucleotide polymorphisms (SNPs), such as g.841G/C (rs41920005) and g.17924A/G (rs41919985), was conducted. The SNP g.841G/C (the conventional designation for this SNP was adopted as SNP 1) is located in the 1st exon of the FASN gene [8,21]. Conversely, the SNP g.17924A/G (the conventional designation for this SNP was adopted as SNP 2) is located in the 39th exon of the FASN gene [21,40]. The SNP g.841G/C is referred to as the SNP g.763G/C in some publications [8,41].

The amplification of the isolated DNA fragments was carried out using a real-time polymerase chain reaction (PCR). Real-time PCRs with high-resolution melting (HRM) analyses were carried out using the HRM master mix—SensiFAST^TM^ HRM kit (Bioline Ltd., Meridian Bioscience, London, UK) and the Real-Time 7500 Fast thermocycler (Applied Biosystems, Foster City, CA, USA). The composition of the PCR mixture (20 μL) for all SNPs was optimized as follows: 10 μL of Master Mix (SensiFAST HRM Mix), 1 μL 10 μM of forward primer, 1 μL 10 μM of reverse primer, 2 μL of DNA solution, and 6 μL of molecular water (nuclease-free) [38].

The sequences of primer pairs for each SNP were as follows:

SNP1:For:5′-CCGACTCGCAACTTCCGAC-3′;Rev:5′-CTCCACCGCACACTCCATC-3′,

SNP 2: For: 5′-ACACGGCTCAACTCGGT-3′;Rev: 5′-TGACATACCTCCTGTAC-3′.

**Figure 1:**
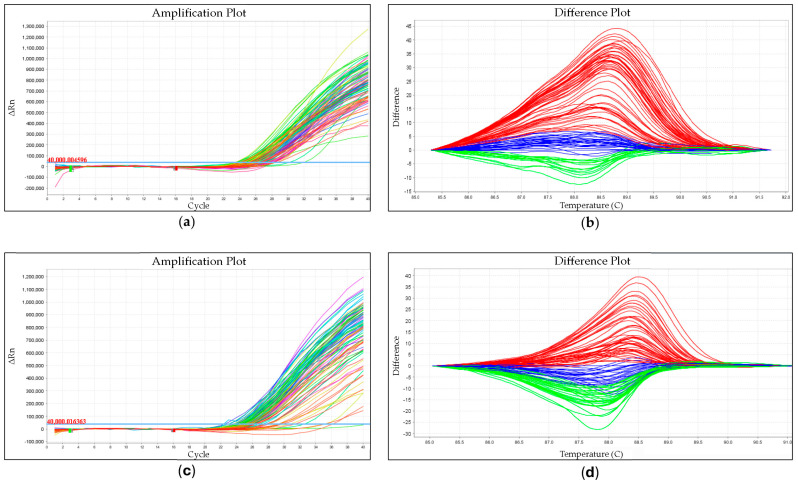
An example of the amplification diagram of the test samples for SNP 1 (**a**) and SNP 2 (**c**) and an example of the high-resolution melting profile (HRM) of the amplicons of the tested samples for SNP 1 (**b**) and SNP 2 (**d**). (**b**): Red is used for CC homozygotes, blue for C/G heterozygotes, and green for GG homozygotes. (**d**): Red is used for GG homozygotes, blue for G/A heterozygotes, and green for AA homozygotes.

The EvaGreen^®^ fluorophore was used for the HRM analyses. The temperature and time conditions of amplification (Figure 1a,c) were optimized as follows: initial denaturation (95 °C for 3 min) and 45 cycles of PCR (denaturation: 95 °C for 10 s; primer hybridization: 65 °C for 10 s; elongation: 72 °C for 15 s). After the elongation stage, the fluorescence was recorded in the FAM channel. The temperature and time conditions for the amplicons’ melting profile analyses were as follows: 95 °C for 15 s, 65 °C for 15 s, and 95 °C for 0.5/5 s. Fluorescence is measured as the sample is heated. After the denaturation of the double-stranded DNA, a sharp decrease in fluorescence was recorded. DNA length and base pair content influenced the recorded melting temperature. Based on the high-resolution melting profiles (HRM) analyses of the amplicons, three genotypes were identified for each SNP. The shape of the melting curve indicates a specific genotypic variant [38] (Figure 1b,d).

Based on the protocol included with the HRM master mix kit, the SensiFAST^TM^ HRM kit (Bioline Ltd., Meridian Bioscience), genotypic variants were determined for each SNP using differential melting curve patterns for the individual genotypes. The principle of SNP genotyping, which involves comparing the melting curves of their genotypes, was described in a previous work [38].

### 2.4. Statistical Analysis

The normality of the distributions of the variables was verified using the Kolmogorov–Smirnov test. A multivariate analysis of variance and Bonferroni’s post-hoc multiple comparison tests were used to verify the significance of the differences.

To compare the observed frequencies with the expected frequencies, assuming the null hypothesis that there is no relationship between the two variables, Pearson’s chi-square (χ^2^) test was used. In addition to determining whether a relationship existed between the variables using the chi-square statistic (χ^2^), the strength of the relationship was also assessed using Cramer’s V coefficient (Vc). A significance level of 0.05 (*p* < 0.05) was assumed for all analyses.

In subsequent analyses, relationships between breed and SNP 1 genotype were examined to determine whether they could significantly differentiate the results of the dependent variables (fat content and individual milk fatty acids). For this purpose, an analysis of variance for factorial systems was used. The significance of the differences (*p* < 0.05) and the strength of the effect (fractional eta-squared (η²) and Fisher’s (F) test) for the relationships were determined. After identifying the significant differences (*p* < 0.05) for specific dependent variables between at least 2 study groups, Bonferroni’s post-hoc multiple comparison tests were used to pinpoint those groups with statistically significant differences (*p* < 0.05). Relationships between breed and SNP 2 genotype were analyzed in the same manner. All the analyses were performed using the Statistica v.13.1 software package. Similar analyses were described previously by Przybylska and Kuczaj [38].

## 3. Results

### 3.1. Frequency of SNP Genotypes of the FASN Gene

The frequency of genotype manifestation for each SNP is presented in Table 1. In the case of the SNP g.841G/C, it was shown that the CC genotype appeared most frequently in the tested animals (RP—0.66; ZR—0.63; RW—0.55). The frequencies of the SNP g.841G/C genotypes only differed significantly between the RP and ZR breeds in the case of the CC genotype (*p* < 0.05). The GG genotype was the least frequent SNP 1 genotype observed across all the breeds studied, with occurrence rates of 0.05 in ZR, 0.07 in RP, and 0.18 in RW. The frequency of the GG genotype was significantly lower in RW cows compared to other groups (*p* < 0.05). There were no statistically significant differences between the breeds of the cows in relation to the frequency of the G/C genotype.

For the SNP g.17924A/G, the GG genotype was most frequent in the RP and ZR cows, with frequencies of 0.82 and 0.73, respectively (*p* < 0.01). Conversely, RW cows exhibited the highest frequency of the A/G genotype at 0.30 (*p* < 0.01). The AA genotype of SNP 2 had the lowest frequency across all breeds, with frequencies of 0.20 in RW, 0.13 in ZR, and 0.05 in RP. However, these differences were not statistically significant. In addition, all the breeds included in our study demonstrated high levels of homozygosity (*p* < 0.01), indicating low genetic diversity.

#### 3.1.1. Effect of the SNP g.841G/C

Bonferroni’s post-hoc tests did not show statistically significant differences in fat concentration between the genotypes of the SNP g.841G/C of the breeds studied (Table 2). However, significant (*p* < 0.05) and highly significant (*p* ≤ 0.01) differences were noted in the contents of some SFAs (C6:0, C8:0, C10:0, C12:0, C14:0, C16:0,andC17:0) and UFAs (C18:1n9c, C18:1n9t,C18:1n7t, C18:2n6c, CLA, C18:3n3, and C20:1),as well as in the total contents of SFAs and UFAs (Table 2) between the genotypes of the SNP g.841G/C.

RW cows with the GG genotype had the highest levels of caproic acid (C6:0) and caprylic acid (C8:0), measuring 0.99% and 0.83%, respectively. These quantities were significantly (*p* < 0.05 for C6:0) and highly significantly (*p* < 0.01 for C8:0) higher compared to RP cows with the G/C SNP 1 genotype, which exhibited 0.76% C6:0 and 0.62% C8:0. Additionally, the C8:0 level in milk fat from RW cows with the GG genotype was highly significantly (0.83%; *p* ≤ 0.01) higher than in RP cows with the GG genotype (0.54%). Moreover, RW cows with the CC genotype had highly significantly and significantly more caprylic acid in their milk fat than RP cows with the G/C (*p* ≤ 0.01) and GG (*p* < 0.05) genotypes, respectively. The three highest C8:0 acid contents were determined to be in the milk fat of RW cows and measured 0.83% for the GG genotype, 0.79% for the CC genotype, and 0.78% for the GC genotype, respectively. The largest number of significant (*p* < 0.05) and highly significant (*p* ≤ 0.01) differences in C8:0 acid content were found between the milk fat of RW and RP cows. ZR cows with the CC and G/C genotypes was characterized by significantly (*p* < 0.05) higher C8:0 acid contents, amounting to 0.75% and 0.77%, respectively, compared to RP cows with the G/C genotype, the milk fat from which had a C8:0 acid content of 0.62%.

The highest amount of capric acid (C10:0) was recorded in the milk fat from RW cows with the GG genotype variant (2.34%). Significantly (*p* < 0.05) and highly significantly (*p* ≤ 0.01) higher amounts of this acid were found in the milk fat of RW cows when compared with specific genotypic variants. The lowest amount of C10:0 acid was recorded in the milk fat of RP cows with the GG SNP 1 genotype variant (1.30%). In the case of the conservation breeds (ZR and RP), a statistically significant (*p* < 0.05) difference in the C10:0 acid content was found between the homozygote CC SNP 1 (2.02%) of the ZR breed and, successively, the G/C heterozygote (1.61%) and GG homozygote (1.30%) SNP 1 of the RP breed.

The highest percentages of lauric acid (C12:0) and myristic acid (C14:0), recorded as 3.12% and 11.14%, respectively, were found in the milk fat of RW cows with the GG variant of the SNP 1 genotype. The contents of C12:0 and C14:0 acids were statistically significantly (*p* < 0.05 and *p* ≤ 0.01) higher when each genotypic variant of the RW breed (CC, G/C, and GG) was compared with the following RP breed genotypes: CC, G/C, and GG. There was also a statistically significant difference in the amount of C14:0 acid between homozygous genotypes GG of the RW breed and CC of the ZR breed (*p* ≤ 0.01). Looking at milk fat from the conservation breeds (ZR and RP), it was found that the SNP 1 CC homozygotes of the ZR breed were characterized by a higher content of C12:0 acid by 0.53% (*p* < 0.05), compared to the G/C heterozygotes of the RP breed. In the case of C14:0 acid, no statistically significant differences were noted between the ZR and RP breeds. The RP breed with the GG polymorphic variant turned out to provide milk with the highly significantly (*p* ≤ 0.01) lowest contents of the C12:0 (1.68%) and C14:0 (7.37%) acids.

Another fatty acid that was tested, the content of which turned out to be statistically significantly different between the breeds and their genotypic variants, was C16:0 acid. The highest amount of this acid was found in the milk fat of RW cows with the GG genotype variant (32.98%). Conversely, the lowest amount of C16:0 acid was found in the milk of RP cows and also in the homozygous GG genotypic variant (23.18%). Homozygotes of GG SNP 1 in the RW breed had significantly (*p* < 0.05) and highly significantly (*p* ≤ 0.01) higher amounts of C16:0 acid when compared with as many as seven genotypic variants from the three cattle breeds (Table 2). There were no statistically significant differences in the amounts of C16:0 acid between the conservation breeds, ZR and RP.

The last SFA for which statistically significant differences in its amount in the milk fat were noted between the breeds and their genotypes was margaric acid (C17:0). For the individual SFAs described above, their highest content was most often found in the GG homozygotes of the RW breed. However, in the case of C17:0 acid, its highest concentration was recorded in the GG homozygotes of the RP breed (0.97%). The amount of C17:0 acid was significantly (*p* < 0.05) and highly significantly (≤0.01) higher compared to other genotypes of the examined breeds; only the GG variant of the ZR breed did not statistically significantly differ in the amount of C17:0 acid with the GG variant of the RP breed. The second-highest amount of C17:0 acid in milk fat was also found for GG homozygotes, but it was for the second conservative breed, ZR (0.94%). This concentration was highly significantly (*p* ≤ 0.01) higher than in the CC and G/C variants of the ZR breed. In addition, there were many statistically significant differences in the content of C17:0 acid between the CC homozygote of the RW breed (with the lowest margaric acid level of 0.64%) and five genotypic variants among the three cattle breeds studied (Table 2). Highly significant (*p* ≤ 0.01) differences in the amounts of C17:0 acid were also noted between the ZR breed (CC genotype) and the RP breed (the CC and G/C genotypes).

Among the monounsaturated fatty acids (MUFAs), statistically significant differences were noted for the following acids: elaidic acid (C18:1n9t) and trans 7 oleic acid (C18:1n7t). It was found that in the case of both C18:1n9t and C18:1n7t acids, the highest amounts were found in the milk fat of RP cows with the GG SNP 1 genotype (1.39% and 3.66%, respectively). The lowest amounts of C18:1n9t (0.70%) and C18:1n7t (1.07%) acids were determined to be in the milk fat of RW cows with the GG and CC SNP 1 genotypes, respectively. In the case of elaidic acid, highly significant (*p* ≤ 0.01) and significant (*p* < 0.05) differences in its amounts were found between the RW breed (GG genotype) and the ZR breed (the CC and G/C genotypes) as well as in comparison with the second conservation breed—RP (the G/C genotype). Highly significant differences (*p* ≤ 0.01) in the amounts of C18:1n7t acid were also observed, among others, between specific polymorphic variants of the ZR breed and the RW breed. Highly significant differences (*p* ≤ 0.01) were also observed in the amounts of trans 7 oleic acid, among others, between some genotypic variants of the RP and RW breeds (Table 2). In the case of conservation breeds, significant (*p* < 0.05) differences in the amount of elaidic acid were observed between the homozygotes of CC in the ZR and RP breeds (milk samples from the ZR breed exhibited 0.35% more of this acid). In addition, significant (*p* < 0.05) and highly significant differences (*p* ≤ 0.01) in the content of trans 7 oleic acid were found when comparing specific genotypes of the RP and ZR breeds (in each case, a significantly higher content of this acid was observed in samples from the RP breed).

Among the polyunsaturated fatty acids (PUFAs), statistically significant differences in the amount of C18:2n6 acid (linoleic acid) were observed. The highest amount of this acid was found in the milk fat of RW cows with the CC genotype variant (1.69%). Highly significant (*p* ≤ 0.01) differences in the amount of linoleic acid were observed when comparing the concentration of this acid in the milk fat of RW cows (separately considering each genotypic variant: CC, G/C, and GG) with polymorphic variants such as the CC and G/C variants of the ZR breed and the CC and G/C variants of the RP breed (each time, highly significantly more linoleic acid was observed for the RW breed; *p* ≤ 0.01). The lowest amount of linoleic acid was observed for the ZR breed in the variant of the CC genotype (1.15%). However, no statistically significant differences in the content of C18:2n6 acid were found between the conservation breeds of ZR and RP.

Another polyunsaturated fatty acid (PUFA) that contains 18 carbon atoms in its chain is cis 9 trans 11 conjugated linolenic acid (CLA). The highest amount of CLA was found in the milk fat of Polish Red cows in the genotype variant GG (1.48%). The milk with the second highest abundance of this acid was from the ZR breed, in the homozygous variant CC (1.30%). Conversely, the lowest conjugated linoleic acid was found in the fat from RW cows in all genotypic variants of SNP 1 (CC—0.59%; GG—0.60%; G/C—0.65%). Bonferroni’s test showed highly significant differences (*p* ≤ 0.01) in the amount of CLA by comparing each of the genotypes of the RP breed (CC, GG, and G/C) with all genotypic variants of the RW breed (CC, GG, and G/C) (each time, finding highly significantly more CLA for the RP breed). There were also highly significant differences (*p* ≤ 0.01) in the CLA contents between the CC genotype variant (1.30%) and the G/C variant (0.86%) within the ZR breed. The amount of conjugated linoleic acid was also significantly different (*p* < 0.05) between the ZR and RP conservation breeds, but only in the following combination of SNP 1 genotypic variants: the ZR breed (G/C—0.86%) and RP breed (CC—1.20%).

The next polyunsaturated fatty acid (PUFA) that was studied was C18:3n3 acid (α-linolenic acid: ALA). ALA is classified as an ω-3 (omega-3) acid; C18:3n3 acid belongs to the group of essential unsaturated fatty acids (EUFAs) [42]. It was found that the RP cow breed with its genotypic variants (GG—1.18%; G/C—0.89%; and CC—0.88%) is characterized by the highest amount of C18:3n3 acid. Based on the results of Bonferroni’s test, highly significant (*p* ≤ 0.01) differences in the content of ALA were found when comparing the milk fat of RP cows with a specific genotype (in the GG genotype variant) with specific genotypic variants of the second studied conservation breed—ZR—and with each SNP 1 genotype for the RW breed (with more ALA being observed for the RP breed each time) (Table 2). There were also statistically significant (*p* < 0.05) differences in the amounts of α-linolenic acid between GG (1.18%) and CC (0.88%) homozygotes within the RP breed. It was also noted that among the three cattle breeds studied, the lowest α-linolenic acid content was found in the milk fat of RW cows of all genotype variants: CC, GG, and G/C. As a result of Bonferroni’s tests, highly significant (*p* ≤ 0.01) and significant (*p* < 0.05) differences in the concentration of C18:3n3 acid in the milk of cows of specific polymorphic variants of the RW breed compared with specific genotypes of the ZR breed were observed; in each case, significantly and highly significantly more ALA was observed for the ZR breed (Table 2).

The last measured unsaturated fatty acid (UFA) was eicosenic acid(C20:1), which is classified as a monounsaturated fatty acid (MUFA). The highest concentrations of this acid in the milk fat of GG homozygotes were detected in both ZR cows and RW cows (0.16%). Other genotypes of the RW breed, G/C and CC, were characterized by equally high C20:1 acid contents (0.13% and 0.11%, respectively). Conversely, the lowest amount of eicosenic acid was found in the milk fat of GG homozygotes of the RP cow breed (0.07%). Bonferroni’s post-hoc tests showed that in the case of the homozygous variant of the GG genotype for the RW breed, as many as six highly significant (*p* ≤ 0.01) and significant (*p* < 0.05) differences in the concentration of eicosenoic acid were found when compared with other genotypic variants of the studied cow breeds (Table 2). There were no statistically significant differences in the amounts of C20:1 acid between the milk samples of cows of the conservation breeds ZR and RP.

An analysis of the total SFAs and UFAs (including MUFAs and PUFAs) in the milk fat revealed that SFAs had a higher percentage share compared to the UFA contents of all the breeds included in our study.

The highest amount of all the determined SFAs was found in the milk fat of homozygous cows with the GG genotype (63.98%) of the RW breed. This was highly significantly more (*p* ≤ 0.01) compared to the CC homozygotes of the ZR breed and the homozygotes of CC and GG in the RP breed. The other two genotypic variants of the RW breed (CC and G/C) were characterized by significantly (*p* < 0.05) and highly significantly (*p* ≤ 0.01) higher amounts of all the SFAs tested when compared with specific genotypes from the ZR breed and the RP breed. Conversely, the highest percentage of all determined UFAs was found in samples from homozygous cows with the GG genotype (41.83%) from the RP breed. This was significantly (*p* < 0.05) and highly significantly (*p* ≤ 0.01) higher compared to some genotypes of the ZR breed and the RW breed. The GG homozygotes (39.54%) of the ZR breed were the second highest in terms of UFAs among all fatty acids tested. The lowest amount of all SFAs tested was found in the milk fat of the RP breed (for the GG genotype: 50.91%). However, the milk fat amount for the RW breed (for the GG genotype: 32.36%) turned out to have the lowest value of all UFAs tested (significantly (*p* < 0.05) and highly significantly (*p* ≤ 0.01) less in comparison with specific genotypic variants of the ZR breed and the RP breed. There were no statistically significant differences (*p* < 0.05 and *p* ≤ 0.01) in the total contents of SFAs determined or in the total amount of UFAs determined between the conservation breeds, ZR and RP.

#### 3.1.2. Effect of the SNP g.17924A/G

Bonferroni’s post-hoc tests did not show statistically significant differences in the amounts of milk fat between the genotypes of the SNP g.17924A/G of the breeds studied (Table 3). However, significant differences (*p* < 0.05) were noted in the content of one SFA (C20:0) and two UFAs (C18:1n9t (classified as MUFA) and C18:2n6 (classified as PUFA)) between the genotypes of the SNP g.17924A/G of the studied animals (Table 3).

The highest percentage of eicosanoic acid (C20:0) was found in the milk fat of ZR cows with the AA genotypic variant (0.20%), whereas the lowest percentage was observed in ZR cows with the GG genotypic variant (0.14%). This difference was highly significant.

The highest percentage of elaidic acid (C18:1n9t) was found in the milk fat of ZR cows with the AA polymorphic variant (1.83%). The concentration of C18:1n9t acid was highly significantly (*p* ≤ 0.01) higher for AA homozygotes of the ZR breed compared to the GG variant of the RP breed and the following variants, GG, A/G, and AA, in the RW breed. Moreover, the AA homozygotes of the ZR breed showed significantly (*p* < 0.05) more C18:1n9t acid than A/G heterozygotes of the same breed (Table 3). The lowest elaidic acid content was recorded in the milk fat of RW cows with the A/G genotype variant (0.93%). This concentration was significantly (*p* < 0.05) lower than for cows with GG homozygotes of the ZR breed and significantly lower (*p* ≤ 0.01) than for those with AA homozygotes of the ZR breed. The difference between the highest and lowest C18:1n9t acid contents in milk fat turned out to be highly significant (*p* ≤ 0.01).

In the case of linoleic acid (C18:2n6), its highest concentration was found for all genotypic variants of the RW breed, as follows: GG (1.62%), AA (1.62%), and A/G (1.61%). All these genotypes of the RW breed differed in a highly significant (*p* ≤ 0.01) manner regarding the content of this acid, compared to the homozygous variants of the ZR breed and the GG homozygotes of the RP breed. In addition, it was found that relatively high levels of linoleic acid were found in the A/G heterozygotes of the RP breed—1.46% (significantly more than the GG and AA homozygotes (*p* < 0.05) of the ZR breed and the GG homozygotes (*p* ≤ 0.01) of the RP breed). It is worth mentioning that the polymorphic variant (A/G) of the ZR breed was also characterized by a relatively high concentration of C18:2n6 (1.32%). Conversely, the lowest concentration of C18:2n6 acid was found in the AA homozygotes (0.99%) of the ZR breed. The difference between the highest and lowest amounts of linoleic acid turned out to be highly significant (*p* ≤ 0.01).

In addition, it was noted that the milk fat of the heterozygote A/G of the SNP g.17924A/G of the RP breed was characterized by the highest percentage of all the tested UFAs (at the same time, it was characterized by the lowest amounts of all the tested SFAs). Conversely, the lowest concentration of all the analyzed UFAs was found in the heterozygotes of A/G SNP 2 of the RW breed (at the same time, they were characterized by the highest total amount of all SFAs studied). However, no significant differences were found in the total SFAs and UFAs of the breeds included in our study (Table 3).

The results of our research suggest that by knowing the significant relationships between specific genotypic variants of the SNPs of the FASN gene and the fat content and fatty acids in question, it is possible to increase milk quality by selecting animals with the specific genotypes of certain cattle breeds. Therefore, the SNPs of the FASN gene can be treated as genetic markers for estimating and ultimately improving the quality of milk and meat obtained from cattle (marker-assisted selection).

## 4. Discussion

FASN is a probable/potential candidate gene influencing the fat and fatty acid content of animal milk [8,33]. The SNP g.841G/C found in the FASN gene is located at approximately 51.4 Mbp on BTA 19 [43]. The SNP g.17924A/G is located in the 39th exon of the FASN gene and causes the amino acid threonine to change to alanine [29,32,40]. 

### 4.1. Effect of the SNP g.841G/C

Roy et al. [8] analyzed the genotype and allelic frequencies of the SNP g.841G/C in the fat contents of milk from Holstein–Friesian (HO) cows (the animals were divided into two groups according to their milk fat content: (1) high fat content and (2) low fat content). Elsewhere, Hayakawa et al. [21] determined the genotypic and allelic frequencies of several SNPs of the FASN gene as manifested in a studied population of Japanese Black cattle and Holstein cattle (Tottori Prefecture). Similarly, Kawaguchi et al. [43] analyzed the frequency of the emergence of genotypic variants and individual alleles, among others, of the SNP g.841G/C for two populations of Japanese Black cattle. 

As a result of the research presented in this article, a high frequency of the homozygous genotype CC SNP 1 was found in milk from all the examined breeds (0.66—RP breed; 0.63—ZR breed; 0.55—RW breed) (Table 1). It was observed that in all these breeds, the lowest frequency was shown by the homozygous genotype GG (0.05—ZR breed; 0.07—RP breed; 0.18—RW breed). However, in their analysis of the SNP g.841 G/C, Hayakawa et al. [21] observed that in three populations of Japanese Black cattle and Holstein cattle, the GG genotype was the most frequent (0.82, 0.94, 0.82, and 0.30, respectively), while the CC genotype was the least frequent (0.01, 0.00, 0.01, and 0.20, respectively). Two populations of Japanese Black cattle that were studied by Kawaguchi et al. [43] demonstrated the highest incidence of the GG genotype and the lowest incidence of the CC genotype (in the case of one of the populations studied). In the second study population, the frequency of the CC genotype variant was 0.00. Other studies by Kawaguchi et al. [44], conducted on Japanese Black cattle, confirmed the highest frequency of the GG genotype for SNP g.841G/C (0.843) and the lowest frequency for the CC genotype (0.008). Another source [21], in which the SNP g.841G/C as found in Japanese Black cattle was examined, also confirmed the highest frequency of the GG genotype and the lowest frequency of the CC genotype for the SNP g.841G/C.

After conducting an analysis of variance (ANOVA), Hayakawa et al. [21] noted a statistically significant relationship between the SNP g.841 G/C and the content of C14:0, C14:1, C16:1, and C18:1 fatty acids in the adipose tissue of Japanese Black cattle (*p* < 0.05, *p* < 0.001, and *p* < 0.0001). Similarly, Kawaguchi et al. [43], when analyzing the association of the SNP g.841 G/C with the content of C18:1 (oleic) acid in the adipose tissue of Japanese Black cattle, confirmed the significance of this relationship (*p* < 0.01). However, in the present study, following an analysis of variance, significant and highly significant relationships were found between the SNP g.841G/C and the concentration of larger amounts of the determined fatty acids, namely, C6:0, C8:0, C10:0, C12:0, C14:0, C16:0, C17:0, C18:1n9c, C18:1n9t, C18:1n7t, C18:2n6c, CLA, C18:3n3, and C20:1, the sum of the tested SFAs, and the sum of the analyzed UFAs in the milk fat of cows of three studied breeds (*p* < 0.05 and *p* ≤ 0.01). Bonferroni’s post-hoc multiple comparison tests identified groups of genotypes for the specific breeds, between which there were significant differences in the contents of the abovementioned fatty acids. Only in the case of C18:1n9c were the results of the analysis of variance not confirmed. In the remaining cases, significant (*p* < 0.05) and highly significant (*p* ≤ 0.01) differences in the percentage of these acids were confirmed. 

In a study conducted by Hayakawa et al. [21], significantly higher (*p* < 0.05) levels of C14:0 acid were recorded for the G/C genotype in three populations of Japanese Black cattle and for the CC genotype in Holstein cattle (3.21%, 2.74%, 3.07%, and 2.77%, respectively). Similarly, we found the highest C14:0 acid level for the G/C genotype in the ZR breed (9.72%), although this difference was not statistically significant. For the RP breed, the highest C14:0 acid level was found for the CC genotype (9.28%; *p* < 0.05 and *p* ≤ 0.01 compared to all RW genotypic variants), consistent with the findings of Hayakawa et al. related to Holstein cattle.

In addition, Hayakawa et al. [21] found that the GG genotype variant of the SNP g.841G/C showed significantly lower (*p* < 0.05) acid contents (C14:0, C14:1, C16:0, and C16:1), a lower total content of SFAs tested, significantly more C18:1 acids, and a higher total content of MUFAs tested between genotypes in the first studied population of Japanese Black cattle. In the other two populations of Japanese Black cattle and in the population of Holstein cattle, Hayakawa et al. [21] observed a similar pattern although in the case of some of the fatty acids determined, differences in concentrations between genotypes were not statistically significant. In the research presented in this article, a similarity to the above tendency is noted, primarily in relation to the conservation breeds—ZR and RP. Similarly to the findings of Hayakawa et al. [21], the GG homozygotes of the ZR and RP breeds demonstrated the lowest amounts of the acids C14:0 and C16:0, the lowest total amounts of SFAs (*p* ≤ 0.01 and *p* < 0.05), and the highest concentrations of the following MUFAs from the C18:1 group: C18:1n9c, C18:1n8c (11c), C18:1n9t (only for RP), C18:1n7t (only for RP), and other C18:1 trans acids (only for RP). The above mentioned differences in concentration of these acids were significant (*p* < 0.05) and highly significant (*p* ≤ 0.01) between some genotypes of the cattle breeds studied (Table 2). A pattern was also observed for the RW breed, which was similar to that noted by Hayakawa et al. [21] for the Japanese Black cattle population and the Holstein cattle population. Namely, the CC homozygotes of the RW breed, characterized by the lowest amount of C16:0 acids and the total content of SFAs tested, simultaneously exhibited the highest total contents of UFAs tested, including C18:1n9c, C18:1n8c (11c), C18:1n9t, and C18:1n7t (*p* < 0.05 and *p* ≤ 0.01). The described differences in the concentrations of these acids were statistically significant between specific genotypic variants of the three breeds studied (Table 2).

Kawaguchi et al. [43], on the other hand, observed that both the homozygous variants GG and CC of the SNP g.841G/C had the highest contents of C18:1 acid (slightly more C18:1 acid was found for the CC variant—by 0.56%; *p* < 0.01) in the fat of the studied population of Japanese Black cattle.

Research conducted by Hayakawa et al. [21] indicated the association of the G allele with a higher concentration of unsaturated fatty acids. Based on the above conclusions from the research conducted by Hayakawa et al. [21], Kawaguchi et al. [44] emphasized the importance of the role of the SNP g.841G/C, which, according to the researchers, may play the role of an important genetic marker in terms of improving the dietary value and quality of beef. 

On the one hand, according to Roy et al. [8], the C allele contributes to the higher fat content found in the milk of Holstein–Friesian cows. On the other hand, when looking at the concentration of milk fat in RW cows, the fat content was found to be high for both the CC (4.23%) and GG (4.26%) variants of SNP 1. However, the difference between these genotypes was not statistically significant (Table 2).

### 4.2. Effect of the SNP g.17924A/G

In their study, Abe et al. [29] presented the frequency of the SNP g.17924A/G genotypes by determining the content of C18:1 acids in adipose tissue for Japanese Black cattle. The highest frequency was recorded for variants of the GG genotype, both for the group with a high concentration of C18:1 acids—55.3% (frequency: 0.34)—and for the group with lower C18:1 acids content—47.7% (frequency: 0.32). Similarly, in the studies described in this article, the highest frequencies of the SNP g.17924A/G were observed for the breeds studied: ZR (0.73), RP (0.82), and RW (0.50) (Table 1). However, this is the general frequency of the GG genotype variant for the SNP g.17924A/G in the conducted studies. On the other hand, when considering the total amount of all determined acids from group C18:1 (C18:1n9c, C18:1n8c (11c), C18:1n9t, C18:1n7t, and other C18:1 trans fatty acids) in milk fat for the ZR and RW breeds (Table 3), it was noted that the highest amounts (26.22% and 26.28%, respectively) were found for the AA genotype, despite its lowest frequency being within both the ZR (0.13) and RW (0.20) breeds. In the case of the RP breed, the highest number of all determined acids from the C18:1 group was recorded for the genotypic variant A/G (29.63%), which is the second genotype in terms of the frequency of appearance within the RP breed (0.13) for the SNP g.17924A/G (Table 1). Upon comparing the content of C18:1 acids in milk fat for the ZR, RP, and RW breeds obtained in this study with the amounts of these acids in the adipose tissue of Japanese Black cattle (research by Abe et al. [29]), it was found that they appear in much higher quantities in the meat of the cattle than in the milk of the three breeds studied.

In their research, Schennik et al. [31] indicated a highly significant relationship between the SNP g.17924A/G of the FASN gene with the content of C14:0 (myristic) acid (*p* < 0.001) and the concentration of C18:1n9c (cis 9 oleic) acid in the milk fat of Holstein cattle (Dutch Holstein–Friesian cows). In the case of SNP g.17924A/G heterozygotes, more C14:0 acid (SFA) was found than C18:1n9c acid (UFA). The opposite situation was observed for the GG genotype, which was characterized by a higher amount of cis 9 oleic acid compared to myristic acid. Conversely, in this study, no significant association of the SNP g.17924A/G with the contents of C14:0 and C18:1n9c acids was found in the milk fat of the examined cattle breeds ZR, RP, and RW. Moreover, unlike in the milk of Holstein cattle [31], in the examined cattle breeds, in each genotypic variant of the SNP g.17924A/G, there was more C18:1n9c acid than C14:0 acid. This may be due to differences in the feeding system and may also suggest a greater health-promoting property in milk produced by cows of the studied conservation breeds, ZR and RP, as well as the RW breed, compared to milk from Holstein cows, which were studied by Schennik et al. [31].

Ciecierska et al. [25], analyzing the frequency of the SNP g.17924A/G genotypes of the FASN gene, found that for Polish Holstein–Friesian cattle (PHF), the most common genotypes were A/G heterozygotes (0.52), while AA homozygotes were the least frequent (0.11). Schennik et al. [31] found a similar frequency of emergence of the A/G genotype variant for Holstein cattle (0.50). However, Oh et al. [28] pointed to a significantly lower frequency of A/G heterozygotes (0.25) for Korean cattle (called Hanwoo). They also found a significantly higher incidence of the GG genotype in Korean cattle (0.73). In the case of the Polish Holstein–Friesian Red-and-White breed studied by our team, the GG genotype (0.50) had the highest frequency, while the AA genotype (0.20) was the least frequently observed. The frequency of A/G heterozygotes for the RW breed (0.30) was very close to the frequency of A/G heterozygotes (0.25) that was reported by Oh et al. [28] for Korean cattle (Hanwoo). A very similar frequency of the GG genotype (0.49) compared to the frequency of the GG genotype of the RW breed was also recorded by Zhou et al. [23] for Chinese Holstein cows. The A/G heterozygous variant (0.43) was more common in Chinese Holstein cows than in Polish Holstein–Friesian cows with red -and-white coats (RW).

The ZR breed that we studied was characterized by the same frequency of appearance of the GG genotype variant (0.73), as in the case of the Korean cattle studied by Oh et al. [28]. In our study, it was found that the AA homozygotes of the conservation breeds ZR (0.13) and RP (0.05) showed very similar frequencies of appearance compared with cattle of the PHF breed, as studied by Ciecierska et al. [25], and compared with the Chinese Holstein cows studied by Zhou et al. [23]. The variant of the GG genotype (0.82) appearing in the RP breed had the highest frequency in the present study. 

In the case of an Indonesian Holstein dairy cow population studied by Rahayu et al. [24], the GG genotype (0.63) also turned out to be a genotype that appeared much more frequently than the other genotypic variants of this cow population. A homozygous variant of AA (0.00) was not recorded at all in the studied population of Indonesian Holstein cows. Conversely, the A/G heterozygous variant (0.37) was characterized by a frequency that was similar to the frequency of appearance of the A/G genotypic variant in the RW breed (0.30) and in the abovementioned Chinese Holstein cows (0.43).

Equally low frequencies of the AA genotype of the SNP g.17924A/G have been found by other researchers [40,45]. In a population of Turkish Anatolian red cattle, the frequency of the homozygous AA variant was 0.06 [45]. A slightly higher incidence of the AA genotype (0.08) was found in Chinese Holstein cattle [40]. Conversely, in the case of the research presented in this article, the lowest frequencies of the appearance of AA homozygotes for the SNP g.17924A/G were observed in cows of the RP breed (0.05).

Schennik et al. [31] found that the AA genotype of the SNPg.17924A/G of the Dutch Holstein–Friesian breed was associated with the highest fat concentration and, at the same time, with an increased amount of C14:0 (myristic) acid in milk fat. Conversely, the lower fat content observed in GG homozygotes was associated with a higher percentage of MUFAs in the milk fat of this breed of cow. Ciecierska et al. [25] also found more fat for the AA polymorphism variant of the SNP g.17924A/G of the FASN gene. In the analyses presented in this article, a similarity to the abovementioned research results was noted. Namely, the AA variant genotype of the SNP g.17924A/G (as in the case of the aforementioned authors [23,25,31]) found in both the ZR and RP breeds was associated with the highest fat contents (4.15% and 4.92%, respectively) in the milk of these breeds. Only in the case of the RW breed was the homozygous AA genotype associated with the lowest fat content (4.18%) within the breed. In the case of the RW breed, this was associated with a reduced content of C14:0 acid (10.33%) and an increase in the amount of MUFA (oleic acid: C18:1n9c; 22.06%). It is worth noting that the fat content (4.13%) that was associated with the genotypic variant AA of the Chinese Holstein breed studied by Zhou et al. [23] was similar to the amount of fat determined for the ZR breed and was significantly (*p* < 0.05) the highest among the values for the other variants within the Chinese Holstein breed. Conversely, the variant of GG polymorphism determined a lower fat content (3.82%) within the ZR breed (as observed by other researchers [23,24,31], where the GG homozygous genotype determined a lower amount of fat) and, at the same time, increased the amount of C14:0 acid (9.75%) and resulted in a decrease in the concentration of C18:1n9c acid (20.35%) and C16:0 acid (as reported by Zhang et al. [32] and Oh et al. [28]). In addition, in the present study (for the RP breed), it was observed that the lowest amount of fat (genotype A/G: 4.25%; a similar amount of fat (4.22%) for the A/G variant for Indonesian Holstein cows [24]) within the RP breed was associated with the lowest content of myristic acid and the highest amount of a MUFA (oleic acid). Therefore, the research presented in this article shows that milk from cows of the RW breed (genotype: AA) and cows of the RP breed (genotype: A/G) was characterized by high health-promoting value (a reduced content of fat and C14:0 acid, with an increased content of C18:1 acid, which is important for the health of the consumer). This fact can be exploited in the production of meat [46] and milk [26], making them more desirable to consumers, due to their health-promoting values, by favoring specific genotypic variants of the SNP g.17924A/G of the FASN gene.

Oh et al. [28] indicated a significant relationship (*p* < 0.05) of the SNP g.17924A/G with the contents of SFAs (C14:0, C16:0, and C18:0) and UFAs (C14:1, C18:1, C18:2n6, and C18:3n3) in the adipose tissue of Korean cattle. In the research presented here concerning the milk fat of the breeds ZR, RP, and RW, significant (*p* < 0.05) and highly significant (*p* ≤ 0.01) similarities were found in the content of only C18:2n6c acid (linoleic acid), which plays an important role in human health. Therefore, it can be concluded that SNP g.17924A/G is significantly associated with linoleic acid in both the meat and milk of some cattle breeds.

In addition, it was noted that the frequency of GG homozygotes (0.50) for the RW breed was similar to the frequency of the GG genotype (0.48) for the Chilean Black Frisian cattle studied by Inostroza et al. [26]. Conversely, the frequency of AA homozygotes was the lowest for both breeds (0.20 and 0.10, respectively). For Chilean Black Frisian cattle, A/G heterozygotes (0.43) had a higher frequency of occurrence than heterozygotes for RW cattle (0.30).

According to the literature review, in many breeds of cattle studied, the variant of the GG genotype of the SNP g.17924A/G is the variant that is most often manifested in meat [27,28,46,47,48] and milk [24,26,40]. Additionally, this research confirms the highest frequency of GG homozygotes for all breeds: ZR (0.73), RP (0.82), and RW (0.50). It is worth noting that the frequency of the GG genotype of the RP breed was highly significantly higher (*p* < 0.01) compared to the ZR breed. 

In addition, it is worth noting that the SNP g.17924A/G can be used as a source of important information in an attempt to estimate the fat concentration in cow’s milk [23,24]. Furthermore, as Zhou et al. [23] point out, the A allele of the SNP g.17924A/G could act as a genetic marker that increases the amount of milk fat. 

It should also be mentioned that the A allele of the FASN gene is associated, among others, with a higher milk fat yield in dairy cattle than the G allele [49]. Looking at the amount of fat in the milk of the studied cattle breeds, it was noted that the AA polymorphic variants of both conservation breeds, ZR and RP, were associated with the highest concentration of fat. On the other hand, in the case of the RW breed, the highest fat content was found in the A/G heterozygotes. This information may provide a premise for further research in this area. 

In this research, in all the studied breeds—ZR, RP, and RW— both SNPs of the FASN gene were characterized by high homozygosity values (Table 1), thus suggesting low genetic diversity within the studied cattle breeds. However, heterozygosity is an important factor in the estimation of genetic variation in animals [50].

## 5. Conclusions

This research facilitated the formulation of the following conclusions:

1. The variant of the GG SNP g.841G/C genotype of the FASN gene in the RP breed, whose milk was characterized by the highest fat content, was strongly associated with the highest concentrations of many studied UFAs and the lowest amounts of many analyzed SFAs, indicating the high nutritional value.
2. The variant of the genotype A/G SNP g.17924A/G in the RP breed, whose milk contained the least fat among the other genotypes within the RP breed, was characterized by the highest contents of several tested UFAs and the lowest amounts of many determined SFAs. The polymorphic variants AA and GG in the RW breed were significantly associated with the highest content in the PUFA C18:2n6c, while in the groups of ZR and RP cows, the same was found for the A/G genotype variant. The AA homozygotes of the ZR breed significantly affected the highest amount of the UFA C18:1n9t. All the polymorphic variants of the ZR breed were characterized by the lowest concentration of milk fat among the other studied cattle breeds.

Due to its fat content and fatty acid profile, the raw material obtained from cows of the RP breed with the A/G genotype, the ZR breed with the A/G and AA genotypes, and the RW breed with the AA and GG genotypes is considered the best in terms of its health-promoting properties.

The research results provide a basis for its further continuation and extension. Subsequent studies should consider including larger numbers of cows of the examined breeds. Further analyses of the studied SNPs are necessary to confirm their role in assessing the fat content and fatty acid profile of cow’s milk, thus contributing to improvements in milk quality.

## Figures and Tables

**Table 1:** Frequencies of the genotypes of the SNP g.841G/C (SNP 1) and the SNP g.17924A/G (SNP 2) in the FASN gene for Polish Red-and-White, Polish Red, and Polish Holstein–Friesian Red-and-White breeds.

Breed	*n*	SNP 1 Genotypes			*n*	SNP 2 Genotypes		
ZR	95	CC	GC	GG	93	GG	AG	AA
		63% **	32%	5%		73% **	14%	13%
RP	148	CC	GC	GG	148	GG	AG	AA
		66% **	27%	7%		82% **	13%	5%
RW	227	CC	GC	GG	240	GG	AG	AA
		55%	28%	18% **		50%	30% **	20%
		χ^2^ = 15.92				χ^2^ = 47.28		
		df = 4				df = 4		
		*p* = 0.0031				*p* < 0.00001		
		V_C_ = 0.13				V_C_ = 0.22		

ZR—Polish Red-and-Whitebreed; RP—Polish Red breed; RW—Polish Holstein–Friesian Red-and-Whitebreed; n—number of animals; SNP 1—single-nucleotide polymorphism g.841G/C; SNP 2—single-nucleotide polymorphism g.17924A/G; **—values differ highly significantly between the SNP g.841G/C (SNP 1) and the SNP 17924A/G (SNP 2) genotypes within rows (*p* < 0.01) (frequencies of genotypes considered for each SNP separately); χ^2^—Pearson’s χ^2^ test; df—number of degrees of freedom; Vc—Cramer’s V coefficient.

**Table 2:** Content (mean ± SD) of fat and fatty acids in the milk of Polish Red-and-White, Polish Red, and Polish Holstein–Friesian Red-and-White cows, depending on the SNP g.841G/C (SNP 1) genotypes of the FASN gene.

	ZR				RP			RW	
Trait	Genotype				Genotype			Genotype	
**(%)**	CC (*n* = 60)	GC (*n* = 30)	GG (*n* = 5)	CC (*n* = 98)	GC (*n* = 40)	GG (*n* = 10)	CC (*n* = 124)	GC (*n* = 63)	GG (*n* = 40)
Fat	3.74 ±0.81	4.19 ±0.79	4.19 ±0.77	4.48 ±0.94	4.45 ±0.81	4.71 ±1.02	4.23 ±0.82	4.18 ±0.85	4.26 ±0.90
C4:0	0.69 ±0.39	0.75 ±0.39	0.72 ±0.49	0.81 ±0.42	0.61 ±0.35	0.65 ±0.24	0.72 ±0.30	0.68 ±0.31	0.82 ±0.35
C6:0	0.88 ±0.30	0.94 ±0.34	0.81 ±0.33	0.93 ±0.32	0.76 ^i^ ±0.27	0.72 ±0.17	0.92 ±0.22	0.87 ±0.26	0.99 ^e^ ±0.25
C8:0	0.75 ^e^ ±0.20	0.77 ^e^ ±0.24	0.63 ±0.17	0.72 ±0.22	0.62 ^a,b,G,H,I^ ±0.18	0.54 ^g,I^ ±0.12	0.79 ^E,f^ ±0.16	0.78 ^E^ ±0.18	0.83 ^E,F^ ±0.16
C10:0	2.02 ^e,f^ ±0.55	2.01 ±0.66	1.62 ±0.20	1.85 ^G,I^ ±0.58	1.61 ^a,G,H,I^ ±0.48	1.30 ^a,G,H,I^ ±0.38	2.17 ^D,E,F^ ±0.51	2.15 ^E,F^ ±0.61	2.34 ^D,E,F^ ±0.55
C11:0	0.18 ±0.06	0.14 ±0.05	0.14 ±0.07	0.05 ±0.03	0.04 ±0.01	nm nm	0.34 ±0.84	0.12 ±0.06	0.04 ±0.02
C12:0	2.62 ^e^ ±0.72	2.60 ±0.88	2.06 ±0.32	2.31 ^G,H,I^ ±0.66	2.09 ^a,G,H,I^ ±0.56	1.68 ^G,H,I^ ±0.44	2.84 ^D,E,F^ ±0.74	2.87 ^D,E,F^ ±0.85	3.12 ^D,E,F^ ±0.85
C13:0	0.07 ±0.04	0.07 ±0.03	0.05 ±0.01	0.06 ±0.02	0.06 ±0.02	0.05 ±0.02	0.11 ±0.07	0.11 ±0.06	0.09 ±0.03
C14:0	9.65 ^i^ ±1.76	9.72 ±2.20	8.53 ±1.34	9.28 ^G,h,I^ ±1.81	8.63 ^G,H,I^ ±1.56	7.37 ^G,H,I^ ±1.64	10.44 ^D,E,F^ ±1.68	10.35 ^d,E,F^ ±1.87	11.14 ^a,D,E,F^ ±1.95
C15:0	1.12 ±0.24	1.09 ±0.22	1.22 ±0.38	1.21 ±0.23	1.18 ±0.26	1.39 ±0.18	1.16 ±0.31	1.23 ±0.35	1.36 ±0.31
C16:0	26.38 ^G,H,I^ ±4.03	28.89 ^i^ ±5.69	24.87 ^i^ ±2.90	26.98 ^G,H,I^ ±4.22	26.32 ^G,H,I^ ±3.93	23.18 ^G,H,I^ ±2.16	29.84 ^A,D,E,F,i^ ±4.06	30.11 ^A,D,E,F^ ±4.26	32.98 ^A,b,c,D,E,F,g^ ±4.87
C17:0	0.65 ^C,D,E,F^ ±0.14	0.66 ^C,F^ ±0.17	0.94 ^A,B,G^ ±0.25	0.75 ^A,F,G^ ±0.14	0.77 ^A,f,G^ ±0.13	0.97 ^A,B,D,e,G,H,I^ ±0.14	0.64 ^C,D,E,F,h^ ±0.13	0.72 ^F,g^ ±0.16	0.70 ^F^ ±0.14
C18:0	11.07 ±2.12	11.05 ±2.63	12.19 ±2.37	11.99 ±2.25	12.20 ±2.35	12.88 ±1.07	10.30 ±2.08	10.52 ±2.59	9.56 ±2.34
C20:0	0.15 ±0.06	0.16 ±0.07	0.19 ±0.04	0.17 ±0.05	0.16 ±0.04	0.20 ±0.03	0.16 ±0.05	0.17 ±0.04	0.17 ±0.03
Σ SFA	56.07 ^g,h,I^ ±5.72	58.73 ±8.29	53.77 ±3.05	57.04 ^I^ ±6.96	54.97 ^G,H,I^ ±6.44	50.91 ^g,h,I^ ±3.61	59.72 ^a,E,f^ ±6.28	60.31 ^a,E,f^ ±6.31	63.98 ^A,D,E,F^ ±6.63
C14:1	1.45 ±1.03	1.28 ±0.36	1.39 ±0.47	1.28 ±0.25	1.23 ±0.24	1.28 ±0.28	1.42 ±0.31	1.40 ±0.35	1.55 ±0.22
C16:1	5.53 ±1.55	5.36 ±2.10	5.53 ±1.78	4.85 ±1.92	5.54 ±1.99	5.54 ±0.76	4.79 ±1.63	4.98 ±1.94	4.36 ±1.95
C17:1	0.37 ±0.11	0.38 ±0.13	0.39 ±0.13	0.46 ±0.10	0.45 ±0.10	0.53 ±0.07	0.31 ±0.11	0.36 ±0.14	0.35 ±0.14
C18:1n9c	20.22 ±4.29	20.74 ±3.94	25.43 ±4.59	22.10 ±3.08	22.31 ±4.04	24.04 ±2.71	21.94 ±4.41	21.55 ±4.85	20.24 ±5.35
C18:1n8c (11c)	0.75 ±0.31	0.77 ±0.22	0.84 ±0.17	0.80 ±0.25	0.84 ±0.24	0.86 ±0.19	1.02 ±0.32	0.93 ±0.31	0.80 ±0.32
C18:1n9t	1.36 ^d,I^ ±0.63	1.20 ^i^ ±0.79	1.00 ±0.74	1.01 ^a^ ±0.62	1.25 ^I^ ±0.54	1.39 ±0.35	1.09 ±0.53	1.07 ±0.56	0.70 ^A,b,E^ ±0.43
C18:1n7t	3.30 ^B,G,H,I^ ±1.24	2.17 ^A,D,e^ ±1.04	2.16 ±1.19	3.13 ^B,G,H,I^ ±1.45	3.14 ^b,G,H,I^ ±1.62	3.66 ^G,H,I^ ±0.61	1.84 ^A,D,E,F^ ±1.07	1.77 ^A,D,E,F^ ±0.80	1.52 ^A,D,E,F^ ±0.86
Other *trans* C18:1	0.37 ±0.30	0.33 ±0.25	0.19 ±0.01	0.25 ±0.07	0.25 ±0.13	0.31 ±0.03	0.44 ±0.20	0.36 ±0.10	0.57 ±0.41
C18:2n6c	1.15 ^G,H,I^ ±0.39	1.15 ^G,H,I^ ±0.29	1.47 ±0.26	1.21 ^G,H,I^ ±0.28	1.18 ^G,H,I^ ±0.31	1.55 ±0.31	1.69 ^A,B,D,E^ ±0.35	1.61 ^A,B,D,E^ ±0.29	1.49 ^A,B,D,E^ ±0.31
CLA	1.30 ^B,G,H,I^ ±0.56	0.86 ^A,d^ ±0.46	1.17 ±0.93	1.20 ^b,G,H,I^ ±0.65	1.24 ^G,H,I^ ±0.67	1.48 ^G,H,I^ ±0.19	0.59 ^A,D,E,F^ ±0.29	0.65 ^A,D,E,F^ ±0.33	0.60 ^A,D,E,F^ ±0.21
C18:3n3	0.81 ^F,G,H,I^ ±0.25	0.72 ^F,G,i^ ±0.30	0.85 ^g^ ±0.24	0.88 ^f,G,H,I^ ±0.23	0.89 ^G,H,I^ ±0.28	1.18 ^A,B,d,G,H,I^ ±0.17	0.44 ^A,B,c,D,E,F^ ±0.19	0.57 ^A,D,E,F^ ±0.24	0.51 ^A,b,D,E,F^ ±0.21
C20:1	0.08 ^H,I^ ±0.05	0.09 ^I^ ±0.05	0.16 ±0.01	0.08 ^H,I^ ±0.05	0.08 ^H,I^ ±0.04	0.07 ^I^ ±0.01	0.11 ^i^ ±0.05	0.13 ^A,D,E^ ±0.05	0.16 ^A,B,D,E,F,G^ ±0.06
C20:4n6	0.08 ±0.04	0.10 ±0.06	0.08 ±0.01	0.08 ±0.03	0.08 ±0.03	0.07 ±0.03	0.13 ±0.04	0.12 ±0.05	0.12 ±0.03
C20:5n (*cis*-5,8,11,14,17)	30.07 ±0.02	0.07 ±0.03	nm nm	0.08 ±0.03	0.07 ±0.02	0.07 ±0.02	0.07 ±0.02	0.07 ±0.02	0.06 ±0.03
Σ UFA	36.49 ^i^ ±4.61	34.88 ^f^ ±5.70	39.54 ±2.85	37.25 ^I^ ±4.56	38.39 ^I^ ±4.83	41.83 ^b,h,I^ ±3.35	35.52 ±5.51	35.04 ^f^ ±5.41	32.36 ^a,D,E,F^ ±6.07
SFA+UFA	92.57 ±2.81	93.61 ±3.25	93.31 ±3.43	94.29 ±3.14	93.37 ±2.69	92.75 ±0.87	93.98 ±9.87	95.35 ±2.11	96.34 ±1.88

SFAs—saturated fatty acids; UFAs—unsaturated fatty acids; Σ SFA—total saturated fatty acids; Σ UFA—total unsaturated fatty acids; ZR—Polish Red-and-White breed; RP—Polish Red breed; RW—Polish Holstein–Friesian Red-and-White breed; n—number of animals; a, b, c, d, e, f, g, h, and i—values differ significantly between SNP the g.841G/C (SNP 1) genotypes within rows (*p* < 0.05); A, B, C, D, E, F, G, H, I—values differ highly significantly between SNP g.841G/C (SNP 1) genotypes within rows (*p* ≤ 0.01); *p*—probability; a, A—CC polymorphism variant of the SNP g.841G/C for the ZR breed; b, B—CG polymorphism variant of the SNP g.841G/C for the ZR breed; c, C—GG polymorphism variant of the SNP g.841G/C for the ZR breed; d, D—CC polymorphism variant of the SNP g.841G/C for the RP breed; e, E—GC polymorphism variant of the SNP g.841G/C for the RP breed; f, F—GG polymorphism variant of the SNP g.841G/C for the RP breed; g, G—CC polymorphism variant of the SNP g.841G/C for the RW breed; h, H—GC polymorphism variant of the SNP g.841G/C for the RW breed; i, I—GG polymorphism variant of the SNP g.841G/C for the RW breed; nm—not marked; SD—standard deviation.

**Table 3:** Contents (mean ± SD) of fat and fatty acids in the milk of Polish Red-and-White, Polish Red, and Polish Holstein–Friesian Red-and-White cows, depending on the SNP g.17924A/G (SNP 2) genotypes of the FASN gene.

	ZR				RP			RW	
Trait	Genotype				Genotype			Genotype	
(%)	GG (*n* = 68)	AG (*n* = 13)	AA (*n* = 12)	GG (*n* = 122)	AG (*n* = 19)	AA (*n* = 7)	GG (*n* = 120)	AG (*n* = 72)	AA (*n* = 48)
Fat	3.82 ±0.81	4.01 ±0.97	4.15 ±0.73	4.50 ±0.92	4.25 ±0.82	4.92 ±0.90	4.22 ±0.83	4.27 ±0.76	4.18 ±0.92
C4:0	0.72 ±0.39	0.65 ±0.43	0.61 ±0.30	0.75 ±0.40	0.62 ±0.32	0.90 ±0.61	0.71 ±0.31	0.75 ±0.32	0.81 ±0.34
C6:0	0.91 ±0.32	0.88 ±0.36	0.79 ±0.23	0.89 ±0.31	0.75 ±0.26	0.91 ±0.43	0.91 ±0.22	0.94 ±0.27	0.96 ±0.26
C8:0	0.76 ±0.21	0.76 ±0.23	0.67 ±0.19	0.70 ±0.21	0.60 ±0.20	0.65 ±0.25	0.80 ±0.16	0.81 ±0.17	0.80 ±0.18
C10:0	2.04 ±0.58	2.04 ±0.64	1.73 ±0.57	1.80 ±0.55	1.52 ±0.55	1.55 ±0.53	2.20 ±0.57	2.23 ±0.48	2.19 ±0.56
C11:0	0.17 ±0.06	0.15 ±0.05	0.14 ±0.03	0.05 ±0.03	0.04 ±0.02	nm nm	0.54 ±1.15	0.09 ±0.06	0.10 ±0.06
C12:0	2.66 ±0.76	2.61 ±0.83	2.22 ±0.73	2.28 ±0.64	1.95 ±0.61	1.89 ±0.53	2.89 ±0.83	2.92 ±0.66	2.86 ±0.82
C13:0	0.07 ±0.04	0.06 ±0.02	0.06 ±0.02	0.06 ±0.02	0.08 ±0.03	0.04 ±0.01	0.10 ±0.06	0.10 ±0.06	0.11 ±0.05
C14:0	9.75 ±1.84	9.74 ±2.09	8.95 ±2.11	9.18 ±1.81	8.19 ±1.46	8.26 ±1.70	10.60 ±1.87	10.60 ±1.45	10.33 ±1.92
C15:0	1.11 ±0.23	1.10 ±0.31	1.18 ±0.18	1.21 ±0.23	1.25 ±0.27	1.13 ±0.26	1.21 ±0.32	1.20 ±0.32	1.22 ±0.33
C16:0	27.21 ±4.75	28.48 ±5.84	25.11 ±4.20	26.96 ±4.28	24.72 ±2.75	26.14 ±3.88	30.68 ±4.41	30.66 ±3.74	30.02 ±4.95
C17:0	0.66 ±0.17	0.71 ±0.21	0.68 ±0.12	0.76 ±0.15	0.82 ±0.13	0.76 ±0.10	0.69 ±0.15	0.64 ±0.13	0.66 ±0.14
C18:0	10.76 ±2.30	11.48 ±1.99	12.41 ±2.12	11.88 ±2.23	13.04 ±1.91	12.81 ±2.61	10.20 ±2.30	10.51 ±2.23	10.59 ±2.38
C20:0	0.14 ^C^ ±0.04	0.15 ±0.07	0.20 ^A^ ±0.12	0.16 ±0.05	0.18 ±0.04	0.19 ±0.02	0.17 ±0.04	0.17 ±0.04	0.16 ±0.03
Σ SFA	56.82 ±6.61	58.63 ±8.11	54.65 ±5.66	56.61 ±6.95	53.68 ±4.82	55.20 ±9.13	60.91 ±6.73	61.16 ±5.77	60.57 ±6.87
C14:1	1.44 ±0.99	1.27 ±0.26	1.22 ±0.22	1.28 ±0.24	1.19 ±0.17	1.25 ±0.50	1.44 ±0.34	1.45 ±0.27	1.43 ±0.28
C16:1	5.55 ±1.82	5.08 ±1.56	5.94 ±1.04	5.10 ±1.91	4.98 ±1.84	5.24 ±2.48	4.83 ±1.73	4.47 ±1.83	4.38 ±1.97
C17:1	0.38 ±0.12	0.35 ±0.12	0.36 ±0.09	0.46 ±0.09	0.48 ±0.12	0.53 ±0.13	0.33 ±0.13	0.35 ±0.12	0.34 ±0.11
C18:1n9c	20.35 ±4.31	21.20 ±4.79	20.25 ±2.00	21.87 ±3.17	23.88 ±3.51	23.87 ±4.59	21.39 ±4.95	21.28 ±4.06	22.06 ±4.50
C18:1n8c (11c)	0.78 ±0.27	0.78 ±0.42	0.66 ±0.08	0.79 ±0.24	0.90 ±0.22	0.94 ±0.27	0.94 ±0.32	0.95 ±0.33	0.94 ±0.31
C18:1n9t	1.28 ^g^ ±0.63	1.05 ^c^ ±0.64	1.83 ^b,D,F,G,H^ ±0.82	1.07 ^C^ ±0.58	1.26 ±0.61	1.11 ±0.90	1.01 ^C^ ±0.45	0.93 ^a,C^ ±0.64	0.98 ^C^ ±0.64
C18:1n7t	2.96 ±1.32	2.40 ±1.36	3.15 ±1.01	3.18 ±1.55	3.29 ±1.15	2.61 ±0.91	1.80 ±1.09	1.82 ±0.72	1.83 ±0.99
Other *trans* C18:1	0.37 ±0.32	0.31 ±0.14	0.33 ±0.14	0.25 ±0.07	0.30 ±0.16	0.21 ±0.08	0.43 ±0.22	0.42 ±0.18	0.47 ±0.25
C18:2n6c	1.15 ^e,F,G,H^ ±0.37	1.32 ±0.33	0.99 ^E,F,G,H^ ±0.18	1.18 ^e,F,G,H^ ±0.27	1.46 ^a,C,d^ ±0.36	1.21 ±0.18	1.62 ^A,C,D^ ±0.32	1.61 ^A,C,D^ ±0.34	1.62 ^A,C,D^ ±0.36
CLA	1.21 ±0.60	0.92 ±0.56	1.18 ±0.45	1.23 ±0.65	1.30 ±0.57	1.08 ±0.69	0.60 ±0.31	0.66 ±0.27	0.62 ±0.29
C18:3n3	0.74 ±0.27	0.82 ±0.25	0.96 ±0.24	0.89 ±0.24	0.93 ±0.31	0.93 ±0.26	0.49 ±0.21	0.54 ±0.26	0.48 ±0.20
C20:1	0.08 ±0.05	0.09 ±0.05	0.06 ±0.02	0.08 ±0.04	0.09 ±0.06	0.09 ±0.05	0.13 ±0.07	0.13 ±0.05	0.14 ±0.05
C20:4n6	0.09 ±0.05	0.08 ±0.01	0.09 ±0.03	0.08 ±0.03	0.09 ±0.04	0.08 ±0.02	0.12 ±0.04	0.12 ±0.05	0.13 ±0.04
C20:5n (*cis*-5,8,11,14,17)	30.07 ±0.02	0.07 ±0.01	0.07 ±0.02	0.07 ±0.03	0.08 ±0.02	0.08 ±0.01	0.06 ±0.02	0.07 ±0.02	0.08 ±0.03
Σ UFA	36.02 ±5.00	35.34 ±6.22	36.85 ±3.52	37.34 ±4.77	40.06 ±3.21	39.05 ±5.62	34.74 ±5.94	34.28 ±4.97	35.08 ±5.57
SFA+UFA	92.84 ±2.98	93.97 ±2.87	91.50 ±2.50	93.95 ±2.91	93.74 ±2.77	94.25 ±4.66	95.01 ±6.90	94.37 ±9.48	95.65 ±2.47

SFAs—saturated fatty acids; UFAs—unsaturated fatty acids; Σ SFA—total saturated fatty acids; Σ UFA—total unsaturated fatty acids; ZR—Polish Red-and-White breed; RP—Polish Red breed; RW—Polish Holstein–Friesian Red-and-White breed; n—number of animals; a, b, c, d, e, and g—values differ significantly between the SNP g.17924A/G (SNP 2) genotypes within rows (*p* < 0.05); A, C, D, E, F, G, and H—values differ highly significantly between the SNP g.17924A/G (SNP 2) genotypes within rows (*p* ≤ 0.01); a, A—GG polymorphism variant of the SNP g.17924A/G for the ZR breed; b—A/G polymorphism variant of the SNP g.17924A/G for the ZR breed; c, C—AA polymorphism variant of the SNP g.17924A/G for the ZR breed; d, D—GG polymorphism variant of the SNP g.17924A/G for the RP breed; e, E—A/G polymorphism variant of the SNP g.17924A/G for the RP breed; F—GG polymorphism variant of the SNP g.17924A/G for the RW breed; g, G—A/G polymorphism variant of the SNP g.17924A/G for the RW breed; G—AA polymorphism variant of the SNP g.17924A/G for the RW breed; nm—not marked; SD—standard deviation.

## Data Availability

The database relating to the presented study and confirming the presented research results is not publicly available, for data privacy reasons. A request to share data regarding the described study can be sent to paulinaprzybylska2018@gmail.com.

## Supplementary Materials

The following supporting information can be downloaded at: https://www.mdpi.com/article/10.3390/ani14152268/s1, Figure S1: Numbers of herds and animals of the Polish Red-and-White (ZR) breed covered by the protection of genetic resources in Poland (in the years 2005–2023) [35]; Figure S2: Numbers of herds and animals of the Polish Red (RP) breed covered by the protection of genetic resources in the milk assessment in Poland (in the years 1999–2023) [36].
