# Supplementary figures and images for: The Effects of Two Selected Single Nucleotide Polymorphisms of the Fatty Acid Synthase Gene on the Fat Content and Fatty Acid Profile of Cow’s Milk from the Polish Holstein–Friesian Red-and-White Breed versus Two Polish Red-and-White and Polish Red Conservation Breeds Kept in Poland
Source: Animals (Basel). 2024 Aug 4;14(15):2268. doi: 10.3390/ani14152268 (PMC11311015; doi:10.3390/ani14152268)

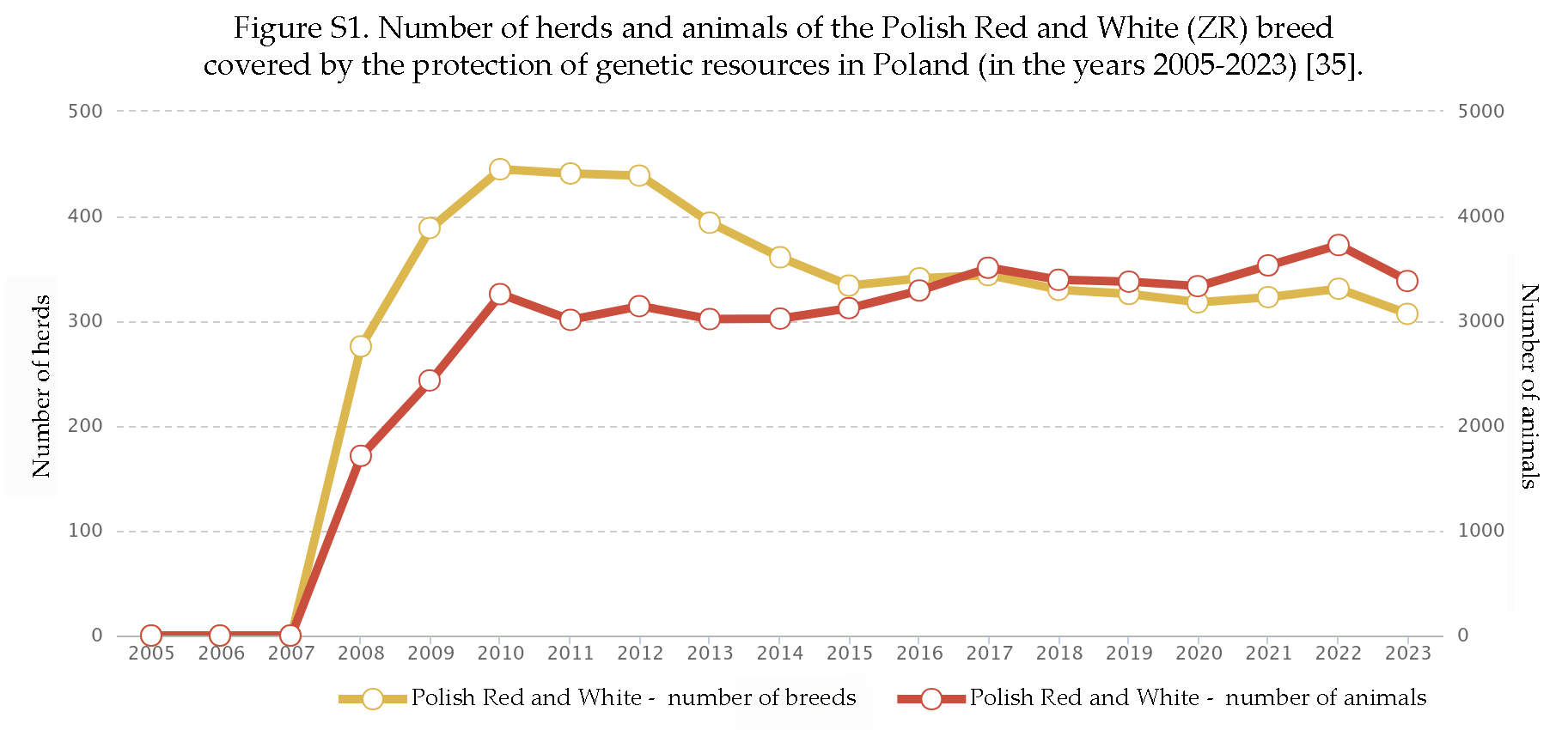

Supplement: Supplementary file 1 [file animals-14-02268-s001.zip › Figure1_[35].jpg]

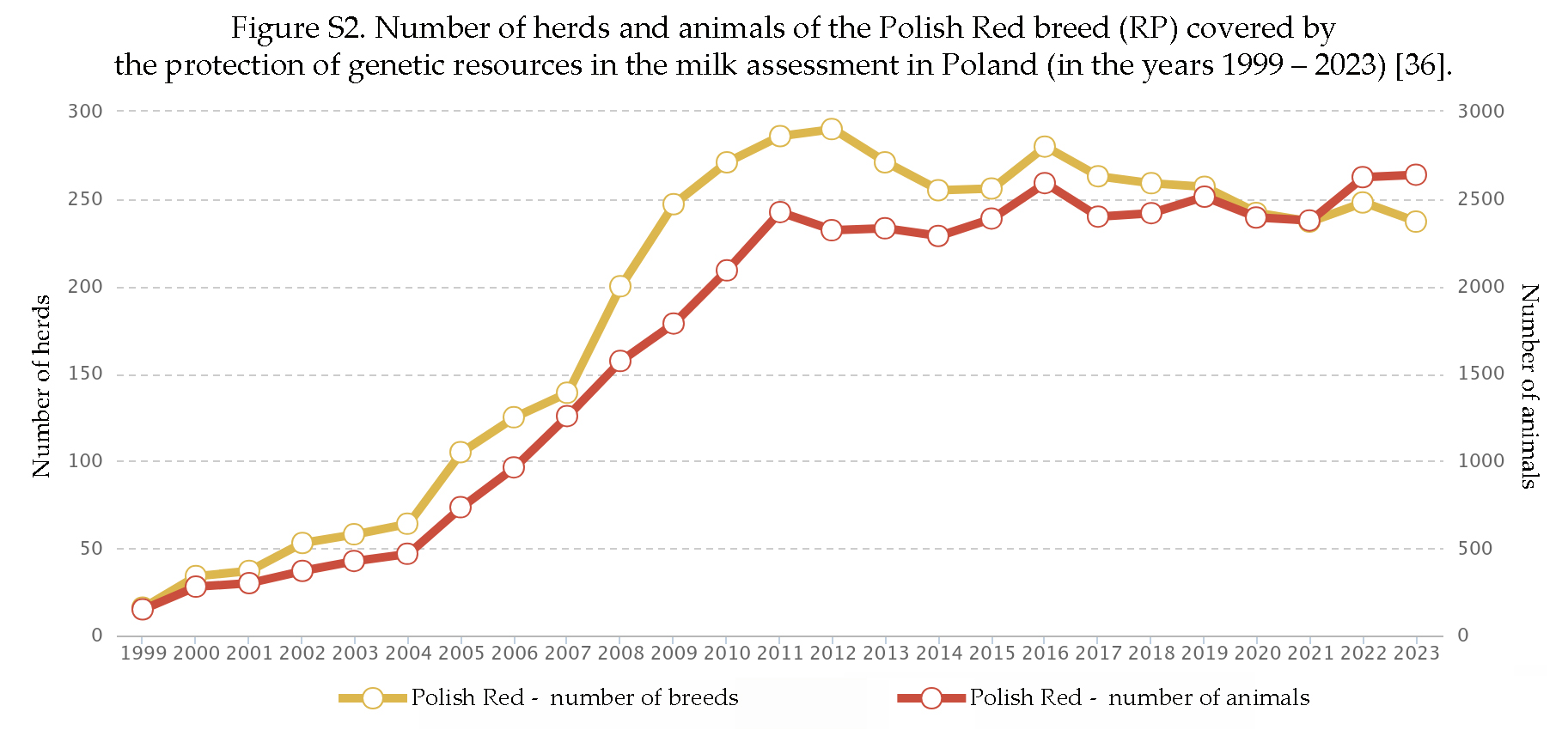

Supplement: Supplementary file 1 [file animals-14-02268-s001.zip › Figure2_[36].jpg]
